# From Pits to Plaques: Electron Microscopy Reveals Clathrin Structural Plasticity in Trafficking, Sorting and Adhesion

**DOI:** 10.1111/tra.70053

**Published:** 2026-08-28

**Authors:** Marion Benoist, Stéphane Vassilopoulos

**Affiliations:** ^1^ INSERM, Institute of Myology, Centre of Research in Myology Sorbonne Université Paris France

## Abstract

Clathrin‐mediated trafficking has classically been described through the formation of curved clathrin‐coated pits and vesicles. However, growing evidence indicates that clathrin assembles into a diverse array of membrane‐associated architectures whose geometry, dynamics, and function vary across cellular compartments and physiological contexts. These include canonical coated pits at the plasma membrane and trans‐Golgi network, extended flat lattices and plaques at the cell surface, clathrin coats associated with endosomal sorting domains, and tubular clathrin assemblies associated with three‐dimensional migration. Here, we synthesize recent work, including cell‐type‐specific, mechanobiological, and ultrastructural studies, to propose clathrin structural plasticity as a core organizing principle of intracellular trafficking. We discuss how adaptor identity, membrane lipid composition, cytoskeletal coupling, and mechanical constraints collectively shape clathrin architectures, enabling a single coat system to support vesicular transport, cargo sorting, signaling, and adhesion.

## Graphical abstract

Clathrin plaques (magenta) form large, flat membrane‐associated lattices embedded within the cortical actin cytoskeleton, and can be distinguished from individual clathrin‐coated pits (yellow). These structures are surrounded by a dense network of actin filaments and associated proteins, illustrating the intimate structural coupling between clathrin assemblies and the actin cortex revealed by electron microscopy.

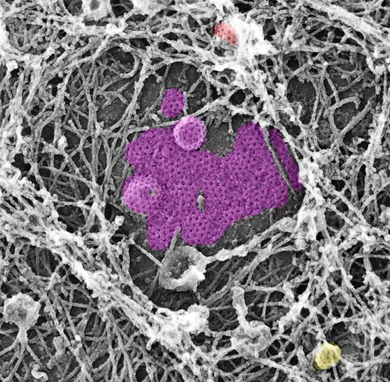

## 1 Introduction

Clathrin is one of the most intensively studied coat proteins in eukaryotic cells and has long been synonymous with receptor‐mediated endocytosis. In the classical view, clathrin triskelia assemble into a polyhedral lattice at the plasma membrane, progressively deforming the membrane into a deeply invaginated clathrin‐coated pit that ultimately undergoes scission to generate a transport vesicle. However, the precise relationship between clathrin assembly and membrane curvature generation remains actively debated, as current models support both progressive curvature acquisition during coat growth and delayed flat‐to‐curved transitions depending on membrane mechanics, adaptor composition, and cytoskeletal coupling [1, 2, 3, 4, 5]. This paradigm, established through decades of biochemical, genetic, and imaging studies, has shaped our understanding of membrane trafficking. Yet, as imaging resolution and temporal sensitivity have improved, it has become evident that this vesicle‐centric model captures only a subset of clathrin‐dependent functions.

A growing body of work has revealed that, in addition to canonical coated pits and vesicles, clathrin can assemble into several structurally distinct membrane‐associated architectures that cannot be explained as simple intermediates of coated vesicle formation. Large flat lattices, stable plaques, extended tubular assemblies, and clathrin coats on endosomes have now been documented in a wide range of cell types and organisms. Importantly, these structures are not sporadic or pathological but appear in specific physiological contexts, including differentiation, migration, and mechanical stress. These observations compel a conceptual shift in which clathrin is viewed not as a uniform vesicle coat but as a structurally plastic scaffold whose organization adapts to the functional requirements of distinct membrane environments.

### 1.1 Structural Plasticity as a Unifying Concept

In this review, we combine ultrastructural observations with recent mechanistic and imaging studies to examine how distinct clathrin architectures support diverse trafficking, adhesion, and membrane‐associated functions across cellular compartments.

### 1.2 Canonical Clathrin‐Coated Pits Across Cellular Compartments

**FIGURE 1:**
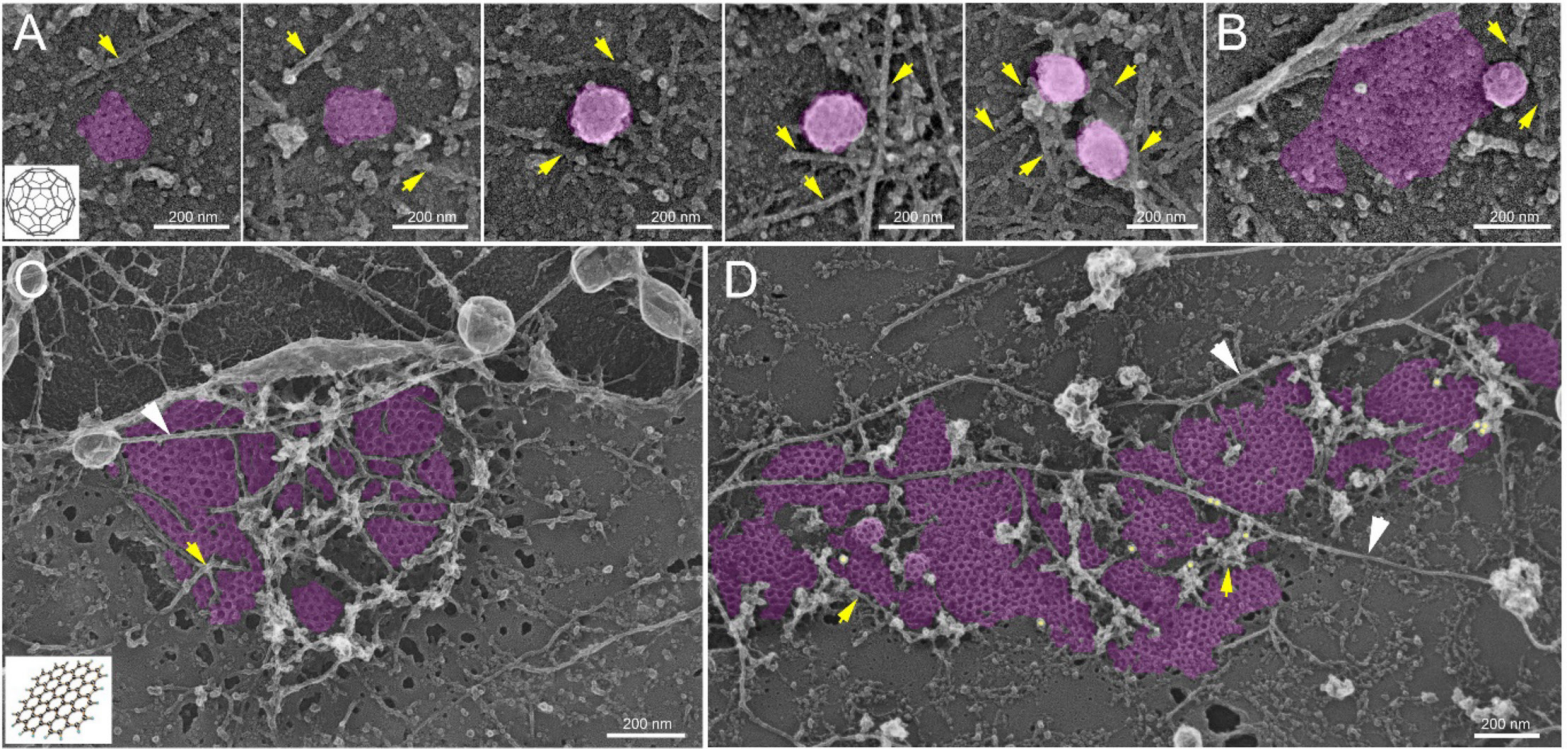
Structural continuum between clathrin‐coated pits and flat clathrin plaques at the plasma membrane. (A) Platinum replica electron microscopy (PREM) images illustrating individual clathrin‐coated pits (CCPs) at different stages of curvature and maturation in Hela cells. Clathrin lattices are highlighted in purple, and arrows indicate associated actin filaments. A schematic representation of a canonical clathrin cage is shown for reference. (B) Example of a CCP connected to a flat clathrin lattice, suggesting a transition between curved pits and planar assemblies in Hela cells. (C, D) Large flat clathrin plaques embedded within dense actin networks in primary mouse myotubes. Plaques are highlighted in purple and display a continuous hexagonal lattice organization. White arrowheads indicate intermediate filaments anchored on branched actin surrounding plaques, whereas yellow arrows mark surrounding branched actin networks. Scale bars: 200 nm in all panels.

Historically, electron microscopy (EM) has played a central role in defining clathrin ultrastructure, with technical advances progressively uncovering increasingly complex clathrin assemblies. Using EM, Roth and Porter discovered clathrin‐coated pits (CCPs) and clathrin‐coated vesicles (CCVs) in their studies on yolk protein uptake in mosquito oocytes [6]. They observed 140 nm ‘bristle‐coated’ pits pinching off from the cell membrane, which then became vesicles that carried the adsorbed yolk protein into the oocyte. Subsequent pioneering work from John Heuser's laboratory combined deep‐etching with rotary shadowing and EM to capture the formation of CCPs in their close‐to‐native state in numerous cell types and tissues (Figure 1A) [7, 8, 9]. Although our knowledge of the roles of clathrin has greatly increased in the past 60 years, the canonical function of clathrin‐coated membrane structures is still understood to facilitate intracellular receptor trafficking. In the case of endocytosis from the cell surface, clathrin is recruited at the plasma membrane (PM) by the heterotetrameric AP‐2 complex (comprising AP2A1 or AP2A2, AP2B1, AP2M1, and AP2S1) [10, 11]. Along with numerous additional partners, clathrin and AP‐2 assemble into CCPs with sizes varying from 60 to 140 nm in diameter that progressively bend the PM to generate receptor‐containing clathrin‐coated vesicles [9, 12, 13]. Importantly, CCPs are not uniform structures but exhibit substantial size heterogeneity across cell types and physiological contexts, with smaller pits typically observed at neuronal synapses and larger assemblies in many non‐neuronal cells [14]. Moreover, cargo occupancy and cargo size can influence coat growth and final pit dimensions [15].

Canonical CCPs remain the most thoroughly characterized clathrin assemblies and serve as a reference point for understanding alternative architectures. At the PM, adaptor protein complex AP‐2 orchestrates the recruitment of clathrin to phosphatidylinositol‐4,5‐bisphosphate(Pi(4,5)P_2_)‐rich domains while simultaneously engaging cargo proteins bearing endocytic motifs [16]. Coat assembly proceeds through stochastic nucleation events followed by lattice growth, curvature acquisition, and eventual scission [14]. Although early models envisioned a largely uniform pathway, it is now clear that pit lifetimes, sizes, and maturation efficiencies vary substantially, reflecting differences in cargo load, membrane tension, and cytoskeletal engagement. These observations support the existence of multiple kinetically distinct clathrin populations, including abortive pits, productive coated vesicles, and long‐lived flat lattices or plaques [15, 17, 18].

Actin dynamics play an important role in modulating canonical pit behaviour. Under conditions of low membrane tension, pits can invaginate largely through clathrin lattice rearrangements and accessory protein activity [19]. By contrast, elevated tension, as encountered in spreading cells or mechanically stressed tissues, increases the requirement for actin polymerization to provide additional force for membrane deformation and vesicle completion [19, 20, 21, 22]. These observations already illustrate the sensitivity of clathrin assemblies to mechanical context, a theme that becomes more pronounced in non‐canonical architectures.

Morphologically similar clathrin‐coated pits operate at intracellular membranes, most prominently at the trans‐Golgi network (TGN). In this compartment, AP‐1 replaces AP‐2 as the principal adaptor, and clathrin recruitment is coordinated by small GTPases such as Arf1 in a phosphatidylinositol‐4‐phosphate (PI4P)‐enriched lipid environment [23]. Golgi‐associated clathrin‐coated vesicles support trafficking toward endosomes and lysosomes, whereas recycling endosomes further expand the repertoire of curved clathrin coats through clathrin‐dependent budding involved in cargo recycling and spatial organization of recycling domains.

Together, these intracellular curved clathrin assemblies establish a transition from canonical plasma membrane endocytosis toward the specialized adaptor‐dependent trafficking pathways that operate at the trans‐Golgi network and endosomal compartments.

#### 1.2.1 Clathrin‐Coated Vesicles at the Trans‐Golgi Network: AP‐1‐ and GGA‐Dependent Sorting Platforms

##### 1.2.1.1 Historical Ultrastructure and Emergence of Golgi‐Associated Clathrin Trafficking

Clathrin‐coated vesicles budding from the trans‐Golgi network represent one of the earliest and most influential examples of intracellular clathrin‐dependent trafficking pathways. Long before the molecular identities of adaptor complexes were established, pioneering EM studies by Marilyn Farquhar and colleagues provided the first evidence that clathrin‐coated vesicles arise from Golgi‐associated membranes and participate in post‐Golgi sorting [24, 25]. These ultrastructural observations were instrumental in shifting the field away from a plasma membrane‐centric view of clathrin and toward a compartmentalized model in which clathrin operates at multiple intracellular sites. EM thus played a foundational role in establishing the trans‐Golgi network as a major hub for clathrin‐mediated trafficking.

##### 1.2.1.2 Adaptor‐Based Specificity and Functional Diversification of Golgi Coats

**FIGURE 2:**
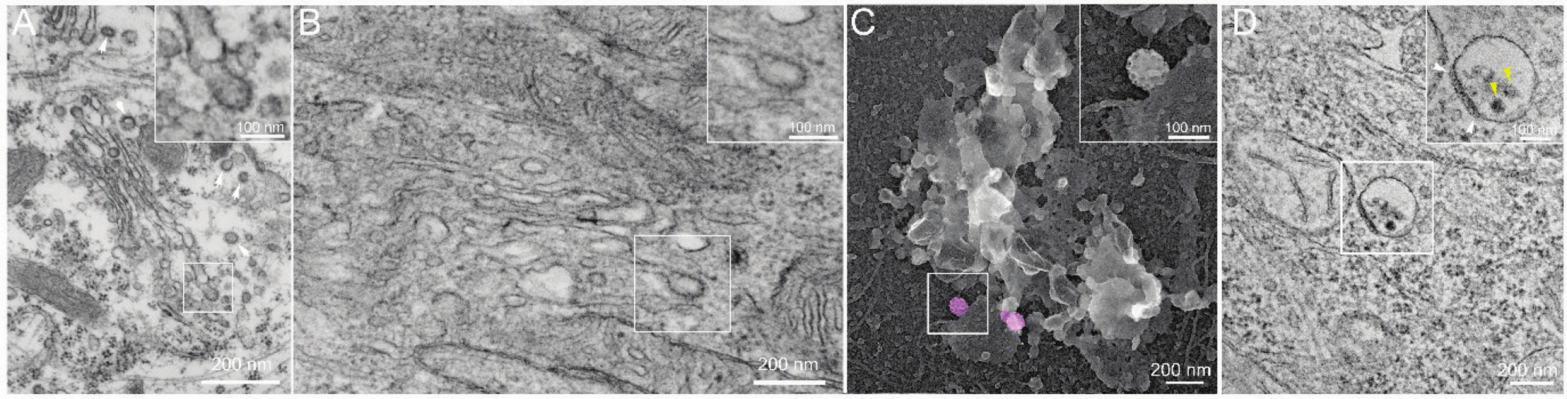
Clathrin‐coated structures at the trans‐Golgi network and on endosomes revealed by thin‐section transmission and PREM. (A, B) Thin‐section transmission electron microscopy (TEM) of the Golgi region from the soma of a mouse spinal cord motoneuron (A) and a cultured myotube (B) showing stacks of flattened cisternae surrounded by numerous small vesicular profiles. Boxed regions are shown at higher magnification in the insets, highlighting coated vesicles and budding profiles with characteristic electron‐dense coats. (C) PREM view of an unroofed Golgi‐associated membrane domain. Clathrin assemblies are highlighted in purple and decorate curved membrane buds and vesicular structures. The boxed region is shown at higher magnification in the inset, illustrating a single clathrin‐coated vesicle with a well‐defined polyhedral lattice. (D) Thin‐section TEM revealing clathrin‐coated endosomal profiles. White arrowheads depict the characteristic bilayered clathrin coat, visible as two electron‐dense layers lining the endosomal membrane, whereas yellow arrowheads indicate intraluminal vesicles. Boxed region and inset highlight the organization of the clathrin lattice on endosomes. Scale bars: 200 nm (main panels) and 100 nm (insets).

Early ultrastructural analyses revealed abundant coated buds and vesicles associated with the trans‐Golgi cisternae and tubular extensions, often positioned at interfaces between Golgi stacks and endosomal compartments (Figure 2) [25, 26, 27]. These studies not only documented the existence of Golgi‐derived CCVs but also emphasized their morphological similarity to plasma membrane‐derived coats, raising early questions about how functional specificity could be achieved using a conserved lattice architecture [11, 25]. Although these structures appeared morphologically similar by EM, later studies showed that their distinct functions are determined largely by adaptor proteins rather than clathrin itself. Trans‐Golgi network (TGN)‐derived clathrin‐coated vesicles also tend to be smaller and more homogeneous than the coated vesicles formed at the plasma membrane [28, 29].

At the TGN, clathrin recruitment is now known to depend on membranes enriched in PI4P and on the coordinated action of the adaptor protein complex AP‐1, monomeric Golgi‐localized γ‐ear‐containing Arf‐binding (GGA1, GGA2 and GGA3) adaptors and the small GTPase Arf1 [30, 31, 32]. AP‐1 plays a central role by coupling cargo selection to clathrin lattice assembly through recognition of sorting motifs within the cytosolic tails of transmembrane proteins [33, 34]. Its recruitment and activation are tightly regulated by Arf1‐GTP, which promotes adaptor association with Golgi membranes, and by phosphoinositide signals that reinforce compartmental specificity [31, 35]. Through these interactions, AP‐1‐dependent clathrin coats mediate transport between the TGN and endosomes. This trafficking is essential for lysosomal enzyme delivery, receptor recycling, and cellular homeostasis [32, 36].

GGA proteins constitute a second major class of clathrin adaptors operating at the TGN and were identified much later than AP complexes, underscoring the long‐standing gap between ultrastructural observation and molecular definition [37, 38, 39]. Unlike heterotetrameric adaptor complexes, GGAs are monomeric proteins that integrate Arf1 binding, phosphoinositide recognition and cargo interaction domains within a single polypeptide [37, 39]. They preferentially recognize acidic dileucine‐based sorting signals found in the cytosolic tails of mannose‐6‐phosphate receptors and related cargos, positioning them as key regulators of lysosome‐directed trafficking [39, 40]. Through their clathrin‐binding motifs, GGAs promote the formation of clathrin‐coated vesicles specialized for cargo export from the TGN toward endosomal compartments [37, 41].

Although AP‐1 and GGAs can independently recruit clathrin, EM and biochemical studies indicate that they often coexist within the same clathrin‐coated domains. Their cooperative assembly enables combinatorial cargo selection and fine‐tunes vesicle composition [29, 39]. This provides a molecular explanation for the diversity of Golgi‐derived clathrin‐coated vesicles that was originally inferred from ultrastructural observations [32, 42]. In this context, clathrin functions as a common structural scaffold, whereas adaptor proteins determine cargo selection and trafficking routes [11, 43]. As a result, vesicles with distinct cargo repertoires and destinations can arise from morphologically similar coats.

Consistent with this specialization, live‐cell imaging studies have shown that AP‐1‐ and GGA‐associated intracellular clathrin coats behave differently from AP‐2‐mediated endocytic coats at the PM. These differences likely reflect the distinct membrane environment and cargo‐sorting requirements of the TGN [44, 45].

#### 1.2.2 Isoform‐Specific Clathrin Pathways at the Golgi

Beyond adaptor diversity, functional specialization at the trans‐Golgi network is further expanded by clathrin isoform usage. Brodsky and collaborators showed that the clathrin heavy‐chain isoform CHC22, encoded by *CLTCL1*, is enriched in muscle and adipose tissues and cooperates with AP‐1 and GGA adaptors to generate GLUT4 storage compartments (GSCs) [46, 47]. Through this isoform‐specific clathrin pathway, GLUT4 is efficiently sorted away from constitutive secretory and endosomal recycling routes and sequestered into a specialized post‐Golgi reservoir composed of long‐lived intracellular vesicles. These CHC22‐dependent GLUT4 carriers are retained under basal conditions and rapidly mobilized to the PM upon insulin stimulation, thereby coupling TGN clathrin‐mediated sorting directly to insulin sensitivity and systemic glucose homeostasis. The GLUT4 reservoir is established initially by diversion of newly synthesized GLUT4 from the endoplasmic reticulum‐Golgi intermediate compartment (ERGIC), creating a zone to which released GLUT4 is then returned by CHC22 through the TGN route. At the mechanistic level, CHC22 recruitment to TGN or ERGIC membranes is mediated by two separate adaptor complexes. The TGN recruitment complex involves sortilin, as well as AP1 and GGA, while the ERGIC recruitment requires dual interaction with sorting nexin SNX5 and the vesicle tethering factor p115 [48, 49]. This dual recruitment mechanism targets CHC22 to early secretory and trans‐Golgi membranes, positioning it at sites of GLUT4 sorting.

#### 1.2.3 Structural Plasticity of Golgi‐Associated Clathrin Coats

Although Golgi‐associated clathrin‐coated vesicles adopt a canonical curved morphology, variations in adaptor composition, lattice dynamics and coupling to regulatory factors generate functionally distinct coats [14]. Importantly, the TGN is a highly dynamic membrane environment characterized by extensive tubulation, fission and fusion events, features that were first appreciated through EM [50, 51]. Within this ultrastructural context, clathrin‐coated vesicles represent one of several parallel mechanisms that collectively shape Golgi‐endosome trafficking, reinforcing the value of EM in revealing both the architecture and logic of intracellular transport pathways [27, 52].

Importantly, the study of AP‐1‐ and GGA‐dependent CCVs reinforces the broader theme that clathrin architecture cannot be understood in isolation from adaptor networks and membrane identity. The trans‐Golgi network and ERGIC provide a clear example in which conserved clathrin lattice geometry is repurposed to support multiple trafficking routes through differential adaptor usage and cargo selection [36, 53].

The Golgi apparatus therefore represents a form of adaptor‐driven clathrin plasticity in which functional specialization emerges despite largely conserved coat ultrastructure.

### 1.3 Clathrin Assemblies at Recycling Endosomes: Classical Ultrastructure and Dynamic Organization

Long before the advent of live‐cell super‐resolution microscopy, EM analyses by Geuze, Stoorvogel, and Klumperman established that clathrin is a prominent and functionally significant component of endosomal membranes [54, 55, 56]. These early ultrastructural studies revealed clathrin‐coated buds and vesicles associated with tubular‐vesicular endosomal compartments, challenging the notion that clathrin function is confined to the plasma membrane and trans‐Golgi network [55, 57]. Recycling endosomes represent a major hub in the endocytic system, coordinating the return of internalized cargo to the plasma membrane and regulating the spatial and temporal aspects of receptor availability [58, 59].

#### 1.3.1 Endosomal Clathrin Coats and Tubular Sorting Domains

At recycling endosomes, clathrin‐coated profiles were observed on both vesicular and tubular membrane domains, often in close proximity to sorting nexuses where cargo segregation occurs [56, 60]. These structures were interpreted as intermediates in endosome‐to‐plasma membrane recycling as well as in endosome‐to‐Golgi transport, supporting the idea that clathrin participates in multiple endosomal sorting routes [61, 62]. Importantly, these coated profiles were frequently smaller and more heterogeneous than canonical plasma membrane‐derived vesicles, suggesting that endosomal clathrin assemblies might operate under distinct physical and regulatory constraints.

Subsequent work reinforced the concept that recycling endosomes are not passive conduits but active sorting platforms in which clathrin plays a central organizational role [58, 63]. Clathrin was found on endosomal tubules as well as on spherical buds, where AP1‐containing coated domains generate transport intermediates involved in receptor recycling and sorting [56, 64]. In this context, clathrin appears to organize specialized sorting domains along tubular membranes while facilitating localized cargo (such as the transferrin receptor) concentration and vesicle formation [56, 65].

#### 1.3.2 Gyrating Clathrin and Highly Dynamic Endosomal Assemblies

A particularly influential contribution to this field came from the identification of “gyrating clathrin,” a dynamic population of clathrin assemblies associated with endosomal membranes [66, 67]. Live‐cell imaging and particle‐tracking analyses revealed that these structures undergo rapid and complex movements, including rotational and translational trajectories, together with high coat turnover. This phenomenon distinguishes them from the progressive maturation behaviour of canonical coated pits [68]. These assemblies were proposed to represent transient, highly dynamic clathrin structures that associate with endosomes to facilitate cargo sorting and membrane remodelling.

The concept of gyrating clathrin provides an important bridge between classical ultrastructural observations and modern dynamic imaging. These assemblies are thought to correspond to clathrin‐positive domains associated with recycling endosomes and recycling carriers rather than to canonical budding intermediates. This links dynamic clathrin behaviour to endosomal sorting, rapid cargo recycling, and migratory processes that depend on efficient membrane trafficking [68, 69, 70].

These observations demonstrate clathrin at recycling endosomes as a prime example of how structural plasticity supports function. Recycling endosomes must accommodate continuous flux, bidirectional trafficking, and rapid changes in cargo composition. In such an environment, rigid or long‐lived clathrin assemblies would be maladaptive. Instead, the prevalence of small, dynamic coats and gyrating clathrin structures suggests that endosomal clathrin is tuned toward rapid assembly and disassembly, enabling control of sorting decisions.

Recycling endosomes therefore illustrate a highly dynamic mode of clathrin structural plasticity in which transient and rapidly remodelled assemblies support reversible sorting and continuous cargo flux within a central intracellular trafficking hub.

### 1.4 Bilayered Clathrin Coats on Endosomes

Flat clathrin coats associated with endosomes were first described by Holtzman and Dominitz and later characterized in molecular and structural detail by subsequent studies from the Klumperman and Stenmark groups [71, 72, 73]. These flat clathrin assemblies adopt a distinct bilayered organization that is ultrastructurally and molecularly distinct from clathrin coats at the plasma membrane [73, 74]. They are formed through direct interactions between clathrin and the ESCRT‐0 component Hrs, which binds clathrin via a dedicated clathrin‐binding motif and recruits it to endosomal membranes enriched in phosphatidylinositol‐3‐phosphate [72, 74, 75]. In EM images, these assemblies appear as dense, flat clathrin lattices opposed to the endosomal limiting membrane. They are readily distinguished from plasma membrane plaques by their increased thickness, multilayered appearance and association with endosomal sorting machinery (Figure 2C) [73, 76]. Unlike canonical clathrin‐coated pits, clathrin‐Hrs coats do not participate directly in vesicle budding. Instead, they define specialized sorting microdomains on the endosomal limiting membrane that concentrate cargo destined for lysosomal degradation, including a broad range of ubiquitylated transmembrane proteins and selected non‐ubiquitylated cargoes [76, 77]. Through its ubiquitin‐interacting motifs and additional adaptor interactions, Hrs captures and concentrates cargoes within these endosomal sorting domains, while the associated clathrin lattice stabilizes and spatially organizes these cargo‐enriched regions [75, 76]. Functionally, clathrin‐Hrs coats act upstream of ESCRT‐mediated membrane remodeling, serving as platforms that coordinate cargo clustering with the sequential recruitment of downstream ESCRT components, including ESCRT‐I, ESCRT‐II and ESCRT‐III, which ultimately drive intraluminal vesicle formation [78, 79, 80]. In this context, clathrin's contribution is primarily organizational rather than mechanical: it provides a stable scaffold that promotes efficient cargo handover to the ESCRT machinery without directly inducing membrane curvature or fission [73, 81]. Recent work further demonstrated that Hrs assembles into two‐dimensional condensates on endosomal membranes that nucleate and organize flat clathrin lattices, providing a mechanistic basis for the formation of these specialized sorting domains [82].

Endosomal clathrin coats thus illustrate the remarkable structural versatility of the clathrin lattice. Instead of canonical endocytic budding, they form specialized flat assemblies dedicated to ESCRT‐associated cargo clustering and degradative sorting. This plasticity is even more evident at the plasma membrane, where clathrin can assemble into large adhesive plaques.

### 1.5 Adhesive Flat Clathrin Lattices and Plaques at the Plasma Membrane

In contrast to curved pits, clathrin frequently assembles into extended flat lattices at the PM (Figure 1B–D) [7, 83, 84]. While such lattices can represent transient intermediates during pit formation, accumulating evidence indicates that they often mature into large, stable structures. These stable structures identified as clathrin plaques appear as bona fide cell–matrix adhesion platforms rather than purely endocytic intermediates [3, 83]. Plaques are particularly abundant in cells subjected to sustained mechanical load, including skeletal muscle cells, osteoblasts, and certain epithelial cells, suggesting that mechanical cues favour lattice stabilization over curvature [85, 86, 87].

Importantly, clathrin plaques are not inert accumulations of coat protein but highly organized membrane domains specialized for adhesion and force coupling. They selectively enrich specific integrins, most notably αvβ5, and partially overlap with reticular adhesion structures that mediate stable attachment to the extracellular matrix (Figure 1D) [87, 88, 89]. Unlike focal adhesions (FA), plaques lack many classical adhesion components but nevertheless provide robust anchoring points that link the extracellular matrix to the numerous branched actin filaments surrounding them (Figure 1B–D) [90]. Endocytic activity is not abolished within plaques but is spatially restricted, occurring preferentially at lattice edges where curvature generation is energetically more favourable (Figure 1B). Several studies have proposed that budding events preferentially occur at the edges of flat clathrin lattices and that AP2 adaptors may be enriched within these peripheral regions, potentially reflecting localized sites of cargo concentration and curvature generation [3, 91]. However, the precise nanoscale organization of AP2 within flat lattices and the extent to which edge enrichment represents a general feature of plaque‐associated budding remain incompletely resolved experimentally. Interestingly, recent work has suggested that the branched actin networks associated with clathrin plaques can also mediate cortical microtubule capture, which might regulate turnover of adhesive clathrin plaques [92].

Muscle cells provide a clear example of clathrin plaque functioning as adhesion structures. During myogenic differentiation, clathrin assembles into large αvβ5 integrin‐containing flat lattices at the sarcolemma, the specialized plasma membrane of muscle fibres, where these structures become prominent components of the cortical membrane organization [85, 93]. These plaques are tightly coupled to branched actin and intermediate filament networks, contributing directly to membrane integrity and resistance to contractile stress (Figure 1C,D) [94]. In this context, clathrin acts as a mechanically resilient scaffold that integrates trafficking with cytoskeletal organization, highlighting how structural plasticity can be used to meet tissue‐specific demands [13]. An additional illustration of clathrin's ability to substitute for canonical adhesion systems is provided by clathrin‐associated reticular adhesions, which persist during mitosis when FAs are disassembled [95]. During cell division, the classical integrin‐talin‐vinculin machinery that underpins FAs is largely dismantled, yet cells retain attachment to the extracellular matrix through αvβ5‐integrin‐enriched reticular adhesions that are intimately associated with flat clathrin lattices [95]. These clathrin‐based adhesion structures provide continuity of substrate attachment across mitosis and contribute to the spatial memory of adhesion sites, thereby influencing post‐mitotic spreading and polarity. A related form of clathrin‐dependent anchoring is also observed in cells migrating within three‐dimensional collagen networks, where classical FAs are sparse or absent. In these environments, clathrin assemblies engage integrins and the actin cytoskeleton to generate mechanically resilient attachment sites adapted to complex extracellular geometries.

Thus, flat lattices and plaques represent a mechanosensitive mode of clathrin structural plasticity adapted to adhesion, force transmission, and signalling functions at the cell surface.

### 1.6 Tubular Clathrin Assemblies and Three‐Dimensional Migration

**FIGURE 3:**
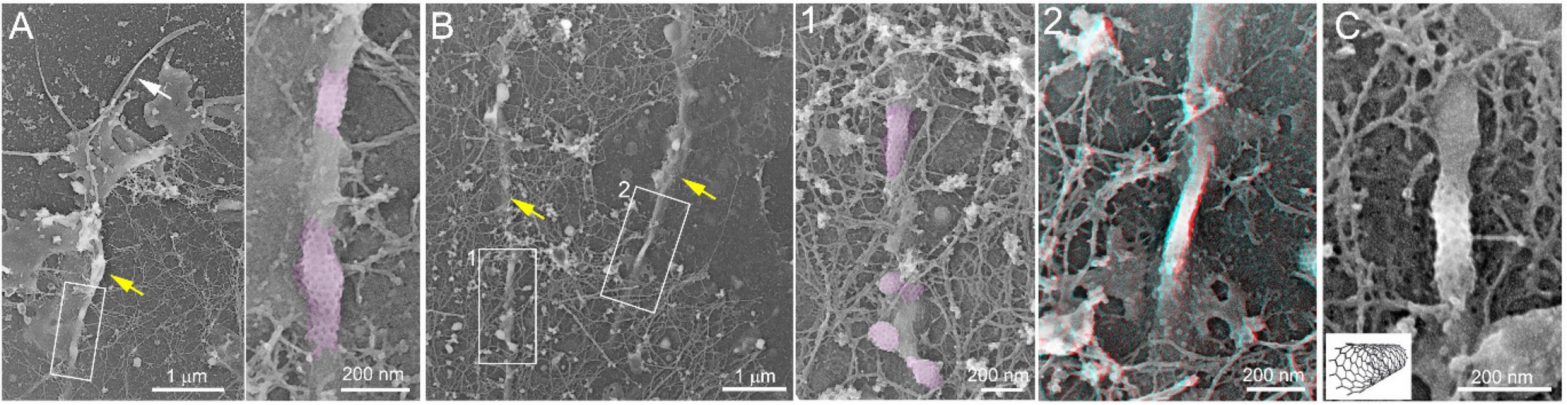
Ultrastructural characterization of tubular clathrin‐AP2 lattices (TCALs). (A–C) PREM views of the cytoplasmic face of the plasma membrane in unroofed MDA‐231 breast cancer cells. (A) Low‐magnification survey view showing an extended tubular membrane structure. Arrows indicate the same collagen fibre either outside (white) or below (yellow) the unroofed cell. The boxed region is shown at higher magnification to the right, revealing a tubular lattice decorated by clathrin assemblies (purple). (B) Survey view of two distinct TCAL‐containing regions; yellow arrows indicate collagen fibres below the unroofed cell, and boxed regions (1, 2) correspond to magnified views shown in panels 1 and 2. Panel 1 shows a clathrin‐decorated tubular structure embedded in the actin meshwork, whereas panel 2 presents an anaglyph view highlighting the three‐dimensional organization of the TCAL (view with red‐cyan glasses). **(C)** High‐magnification view of a TCAL, illustrating its characteristic curved lattice architecture (schematized in inset). Scale bars: 1 μm (A, B), 200 nm (insets and panels 1, 2 and C).

One of the most striking manifestations of clathrin structural plasticity is the formation of tubular clathrin assemblies at the plasma membrane during cell migration in three‐dimensional environments (Figure 3) [96]. In migrating cells, clathrin and the canonical adaptor AP‐2 assemble into tubular clathrin‐AP‐2 lattices (TCALs) at sites where the PM contacts elongated extracellular structures such as collagen fibres (Figure 3A–C) [96]. Rather than generating curved buds or extended plaques, clathrin lattices elongate along the fibre axis, partially wrapping the membrane around the fibril and forming tubular coats. PREM analysis revealed that these clathrin‐coated structures adopt a distinctly tubular morphology and can physically wrap around and pinch collagen fibres. This provides direct ultrastructural evidence that TCALs function as anchoring and force‐transmission elements rather than endocytic intermediates (Figure 3). Consistent with this role, TCALs are enriched in *β*1 integrin, which is required for their assembly, and they promote efficient cell migration within three‐dimensional extracellular matrix networks, including in invasive cancer cell models [96].

Tubular clathrin assemblies illustrate how external geometry and matrix organization can dictate clathrin lattice architecture. Their formation depends on canonical plasma membrane adaptors and phosphoinositide‐rich membranes, yet is strongly modulated by the physical and biochemical properties of the extracellular environment. Notably, the adaptor proteins AP‐2 and Dab2 accumulate at collagen fibre contact sites independently of clathrin, suggesting that adaptor recruitment precedes and templates lattice formation. TCAL assembly is further enhanced by collagen fibre‐associated signaling receptors, such as the epidermal growth factor receptor (EGFR), whose engagement increases the nucleation rate of tubular clathrin structures through interactions with clathrin‐mediated endocytic cargoes [97]. Recent work has expanded this paradigm by showing that TCALs enriched in β5 integrin can also mediate adhesion to tubular membranous material deposited by migrating fibroblasts, facilitating cancer cell attachment and movement along pre‐existing tracks [98]. While tubular clathrin assemblies have been most extensively characterized in cells migrating within three‐dimensional matrix environments, elongated and tubular clathrin morphologies have also been reported in two‐dimensional ultrastructural analyses of cultured osteoclasts, indicating that such architectures are not restricted to 3D migration contexts [99].

Together, these observations demonstrate how elongated extracellular geometries and fibrillar matrix organization can redirect clathrin assembly toward highly tubular membrane architectures. These structures are highly specialized for integrin‐based adhesion, force transmission, and mechanoadaptive migration within three‐dimensional extracellular matrices.

### 1.7 Clathrin Assemblies in Neurons: Dendritic Organization, Axonal Specialization, and Synaptic Recycling

Neurons provide a particularly stringent test for the versatility of the clathrin system because of their extreme polarization and highly extended morphology. They are organised into dendrites, which receive synaptic inputs, the soma containing the major cellular organelles, a long axon responsible for signal propagation, and presynaptic terminals specialized for neurotransmitter release. This complex architecture imposes high demands on spatially and temporally coordinated membrane trafficking, requiring clathrin assemblies to function under conditions that differ markedly from those in non‐neuronal cells. Accordingly, neuronal clathrin has long been recognized as functionally specialized. More recent work has expanded this view, revealing several distinct clathrin architectures that operate in dendrites, axons, and synapses.

#### 1.7.1 Dendritic Clathrin Assemblies and Synaptic Remodeling

**FIGURE 4:**
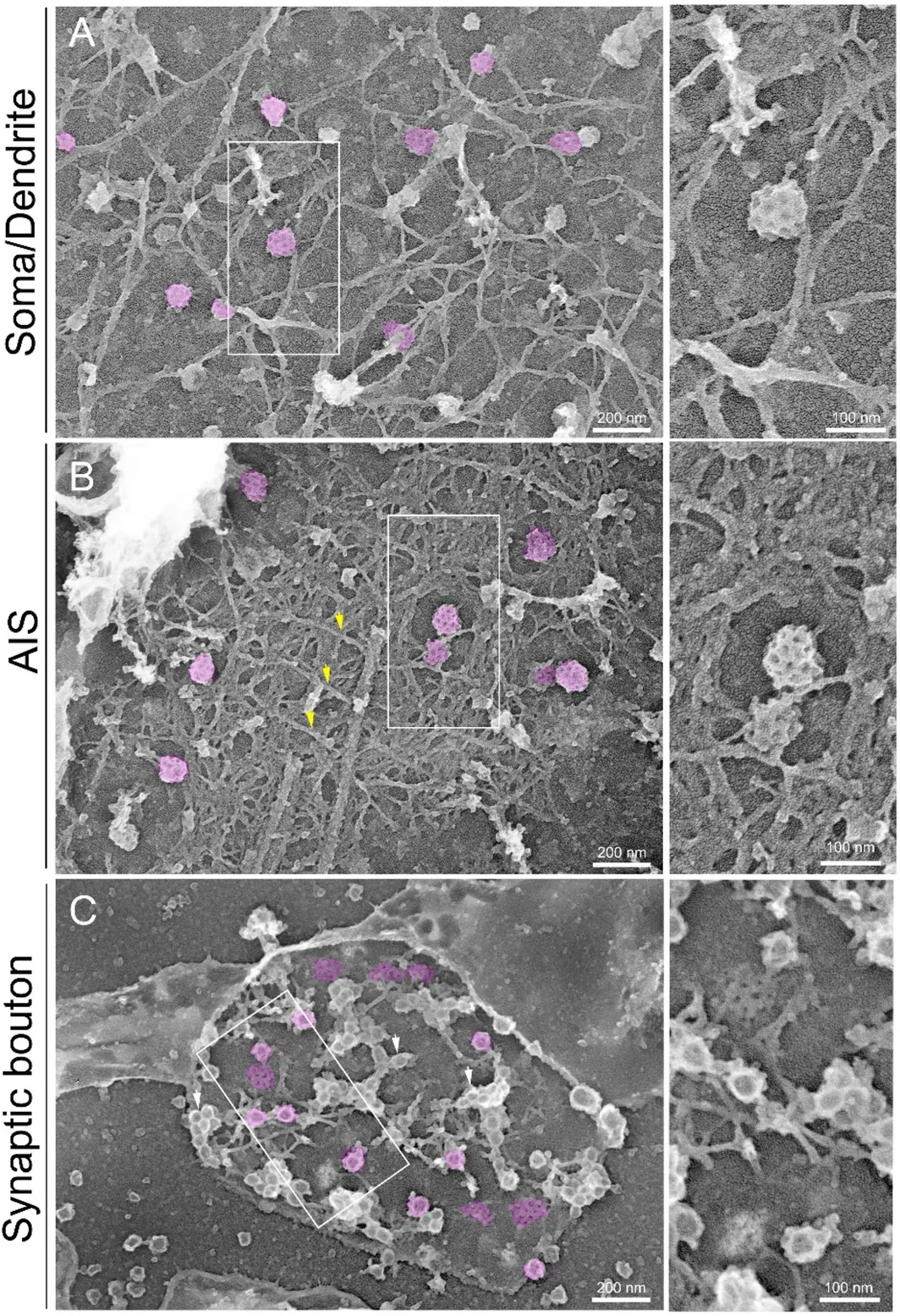
Compartment‐specific organization of clathrin assemblies in neurons revealed by PREM. (A) Low‐magnification PREM view of the somatodendritic plasma membrane showing the distribution of clathrin assemblies (purple) interspersed within an actin‐rich cortical network. The boxed region is shown at higher magnification on the right. (B) PREM view of the AIS membrane displaying a dense cytoskeletal scaffold that defines discrete membrane “clearings” containing stable clathrin assemblies (purple). Yellow arrowheads indicate periodic actin filaments. The boxed region is shown at higher magnification on the right, highlighting clathrin structures that are spatially confined by the AIS cytoskeleton. (C) PREM view of a synaptic bouton enriched in clathrin‐coated pits (purple). The boxed region is shown at higher magnification on the right, revealing small clathrin coats surrounded by synaptic vesicles. Scale bars: 200 nm (main panels) and 100 nm (magnified views).

In dendrites, clathrin assemblies are abundant, where they contribute to receptor trafficking, synaptic plasticity, and dendritic membrane organization. Clathrin‐mediated endocytosis in neuronal soma and dendrites governs the internalization of neurotransmitter receptors, adhesion molecules, and signaling complexes, processes that are central to synaptic strength modulation and circuit remodeling [100]. The role of clathrin in dendritic plasticity is not well established, but clathrin‐mediated endocytosis is necessary for dendritic growth [101]. Interestingly, dendritic endocytosis decreases with neuronal maturation, concomitant with altered clathrin dynamics [102]. In addition to canonical coated pits, dendrites harbour extended clathrin assemblies whose organization and dynamics differ from those observed in axons or somatic regions (Figure 4A) [103, 104]. These assemblies frequently coexist with actin‐rich structures and signaling platforms, suggesting that clathrin participates in local membrane remodeling events coupled to synaptic signaling rather than bulk membrane retrieval. The dendritic compartment therefore highlights an intermediate mode of clathrin function, in which endocytic activity and scaffold‐like roles intersect to support synaptic plasticity.

#### 1.7.2 Stable Clathrin Assemblies at the Axon Initial Segment

Clathrin function in axons has traditionally been viewed primarily through its role in vesicular transport and presynaptic endocytosis. However, this perspective has been substantially revised by recent work identifying a novel and highly specialized clathrin assembly at the axon initial segment (AIS). The AIS is a unique neuronal domain responsible for action potential initiation and for maintaining neuronal polarity by acting as a diffusion barrier between somatodendritic and axonal membranes [105, 106, 107]. Its dense cytoskeletal scaffold and specialized membrane composition were long thought to be incompatible with extensive clathrin involvement. This view was fundamentally challenged by Wernert et al., which demonstrated that clathrin forms particularly stable, long‐lived assemblies at the AIS [108]. These structures are spatially confined to membrane “clearings” delimited by the AIS cytoskeleton and persist for extended periods (Figure 4B). Importantly, these stable clathrin assemblies retain the capacity to be internalized. Stimuli that induce neuronal plasticity trigger their endocytic conversion by stimulating branched actin polymerization around the pits, which generates the curvature needed for vesicle formation. This behaviour reveals a regulated switch between scaffold and endocytic functions, controlled by cytoskeletal remodeling rather than de novo coat assembly. Rather than representing an independent trafficking pathway, AIS‐associated clathrin assemblies appear to preserve the core molecular machinery of canonical clathrin‐mediated endocytosis. Proteomic analyses of the AIS identified numerous endocytic components, including AP2, dynamin, and actin regulatory proteins [109]. This supports the idea that the distinctive behavior of these structures arises from the unique spectrin‐based architecture and mechanical constraints of the AIS rather than from a fundamentally different endocytic mechanism.

#### 1.7.3 Presynaptic Clathrin Coats and Synaptic Vesicle Recycling

At presynaptic terminals, clathrin plays a central role in synaptic vesicle recycling, a process essential for sustaining neurotransmission during repeated rounds of activity. Early ultrastructural analyses of isolated nerve terminals further provided the first compelling morphological evidence for this process, describing synaptic vesicles encased in polyhedral clathrin “baskets” and directly linking coat assembly to membrane retrieval at synapses [110]. Foundational EM studies by Heuser and Reese revealed abundant CCPs and CCVs adjacent to active zones, establishing clathrin as a core component of the synaptic vesicle cycle [111]. Following synaptic vesicle fusion, clathrin‐mediated endocytosis was shown to retrieve membrane vesicles and protein components to regenerate release‐competent vesicles [111]. Subsequent work identified specialized adaptor proteins and accessory factors, such as AP‐2, endophilin, intersectin and synaptojanin, that tailor clathrin function to the unique kinetic and molecular constraints of the synapse [112, 113, 114]. In this context, clathrin assemblies must combine high efficiency with tight regulation, favouring rapidly forming, highly curved coats optimized for fast turnover rather than long‐lived scaffolding. Notably, CCPs at synapses are among the smallest clathrin‐coated pits described to date, likely reflecting the strong geometric and kinetic constraints imposed by rapid vesicle turnover and the need to faithfully regenerate synaptic vesicles of uniform size (Figure 4C). Diversification of clathrin light chain isoforms further contributes to this specialization, as light chain composition directly regulates lattice flexibility and membrane deformation, thereby influencing synaptic vesicle formation efficiency in vivo. Vertebrates express two major clathrin light chain paralogs, CLCa and CLCb, together with neuron‐specific splice variants that differentially regulate lattice mechanics, curvature generation and coupling to the actin cytoskeleton [115, 116]. Recent structural and in vivo studies have shown that neuronal CLC isoforms directly modulate lattice flexibility and membrane deformation efficiency [117], thereby influencing synaptic vesicle formation and neurotransmission [118]. These findings suggest that variation in CLC composition represents an additional mechanism by which clathrin structural plasticity can be tailored to the functional demands of specific cellular compartments. The presynapse thus exemplifies a regime in which clathrin plasticity is biased toward productive endocytic architectures that preserve vesicle identity while supporting rapid recycling.

Together, dendritic, axonal, and presynaptic clathrin assemblies illustrate how neuronal compartments impose distinct spatial and kinetic constraints on clathrin organization. While dendritic and AIS‐associated assemblies support localized membrane remodeling and activity‐dependent stabilization, presynaptic coats are optimized for rapid vesicle recycling and sustained neurotransmission. Neuronal compartments therefore exemplify how clathrin architectures can be selectively tuned to the functional demands of polarized membrane trafficking.

### 1.8 Determinants of Clathrin Structural and Functional Plasticity

The coexistence of curved pits, flat plaques, bilayered coats, and tubular lattices raises a central question: how can a single protein system generate such diverse architectures? A unifying model emerges when clathrin assembly is viewed as a multiscale process regulated by adaptor identity, membrane composition, mechanical forces, and cytoskeletal interactions rather than as a collection of independent assembly pathways. Importantly, these determinants do not act independently but are extensively interconnected, collectively shaping the energetic landscape of coat assembly. Clathrin structural plasticity therefore emerges from the integration of molecular and physical inputs rather than from distinct assembly programs.

#### 1.8.1 Adaptor Identity and Membrane Lipid Composition

Adaptor proteins not only recruit clathrin to specific membranes but also influence lattice stability and curvature propensity. Adaptor composition and clathrin‐adaptor stoichiometry help determine whether lattices remain flat or transition to curved states, with adaptor binding surfaces and dynamics biasing assembly outcomes [119].

Membrane lipid composition provides an additional layer of regulation. Rather than serving solely as membrane landmarks, phosphoinositides actively control the initiation, maturation, and remodeling of clathrin assemblies, acting as central determinants of membrane identity and protein recruitment [120, 121]. At the plasma membrane, PI(4,5)P_2_ is essential for the recruitment and activation of AP2 and numerous endocytic accessory proteins, thereby promoting clathrin‐coated pit initiation, stabilization, and maturation [121, 122]. Dynamic phosphoinositide conversion further accompanies coat maturation and trafficking progression, contributing to the temporal coordination of adaptor turnover and vesicle formation [123]. At the trans‐Golgi network, PI4P‐rich membranes preferentially recruit AP1 and GGA adaptors, establishing a biochemically distinct platform for clathrin assembly and cargo sorting [31, 45]. In contrast, PI3P‐enriched endosomal membranes recruit the ESCRT‐0 component Hrs, which in turn binds clathrin and promotes the formation of specialized flat clathrin coats that organize endosomal sorting domains [72, 75]. Beyond adaptor recruitment, membrane lipid composition can also influence the physical properties of the membrane and the local organization of endocytic proteins, thereby modulating the conditions under which clathrin lattices assemble and remodel [120].

#### 1.8.2 Mechanical Stress, Membrane Tension and Curvature Generation

Mechanical stress and membrane tension constitute major physical determinants of clathrin architecture. Biophysical and live‐cell imaging studies demonstrated that membrane tension strongly influences whether clathrin assemblies undergo productive budding or remain in extended flat states. Under conditions of low tension, canonical clathrin‐coated pits can invaginate largely through lattice rearrangement and accessory protein activity. By contrast, elevated membrane tension increases the energetic barrier for membrane bending and creates a stronger requirement for actin‐mediated force production during pit maturation [19, 22]. Consistent with this view, inhibition of actin polymerization selectively impairs clathrin‐mediated endocytosis under high‐tension conditions, leading to stalled or enlarged coats [19]. Clathrin‐mediated endocytosis is therefore intrinsically mechanosensitive, with higher membrane tension favouring extended flat states and lower tension facilitating curvature. Experimental manipulation of membrane tension alters the balance between flat clathrin lattices and invaginated pits, underscoring the role of mechanical context in structural plasticity [119]. Increased membrane tension can also promote the formation of unusually large and long‐lived clathrin assemblies, including giant coated pits and flat plaques, which require extensive actin networks for internalization or stabilization [21, 84]. Consistent with this view, high‐resolution live‐cell imaging of clathrin and membrane topography demonstrates that curvature can arise either concomitantly with clathrin accumulation or via a delayed flat‐to‐curved transition. This observation supports a flexible model in which multiple productive pathways coexist rather than a single obligatory assembly sequence [5, 124]. Together, these studies support a model in which membrane tension and cytoskeletal force balance act as central regulators of clathrin structural plasticity.

#### 1.8.3 Cytoskeletal Coupling and Force Generation

Cytoskeletal coupling provides active forces that can either drive curvature at lattice edges or stabilize extended flat assemblies. Actin polymerization and actin‐adaptor interactions can promote curvature and vesicle scission, whereas sustained cytoskeletal contacts help sustain large, flat plaques, linking structural organization to force generation and remodeling [91, 92]. Recent super‐resolution analyses further revealed that branched actin networks are asymmetrically organized around clathrin‐coated pits. This asymmetry provides directional force production that facilitates membrane invagination and vesicle formation, highlighting the sophisticated spatial coupling between clathrin lattices and the actin cytoskeleton during endocytosis [125]. More generally, interactions between clathrin assemblies, branched actin networks, adhesion complexes, and mechanosensitive signaling pathways support the idea that clathrin coats function not only as trafficking intermediates but also as mechanically responsive membrane scaffolds.

#### 1.8.4 Genetic, Isoform‐Specific and Evolutionary Determinants

Genetic and post‐transcriptional mechanisms further tune clathrin behavior in a cell‐type‐ and differentiation‐dependent manner. Alternative splicing of the clathrin heavy chain gene (*CLTC* exon 31) has been shown to bias coat organization, with inclusion promoting flat plaque assembly and exclusion favouring curved pits. This isoform‐level control demonstrates that clathrin's intrinsic assembly properties can be genetically modulated to determine structural outcomes and thereby influence tissue‐specific functions and membrane domain organization [13].

At synapses, diversification of neuronal clathrin light chain isoforms (n*CLTA* and n*CLTB*) through alternative splicing provides an additional layer of regulation, tuning lattice flexibility and membrane deformation to optimize synaptic vesicle formation and recycling, and further expanding the molecular basis of clathrin plasticity [118]. Extending this concept across evolutionary scales, comparative analyses in fungi reveal a mosaic evolution of clathrin‐mediated endocytosis. Relatively modest changes in protein composition, assembly order, or actin regulation produce pronounced differences in endocytic dynamics and morphology, underscoring that clathrin structural plasticity is an evolvable trait rather than a fixed core mechanism [126].

Together, these observations support the view that distinct clathrin architectures emerge from the integration of adaptor composition, membrane mechanics, cytoskeletal coupling, and compartment‐specific cellular constraints rather than from intrinsically separate assembly mechanisms.

## 2 Conclusions and Perspectives

**FIGURE 5:**
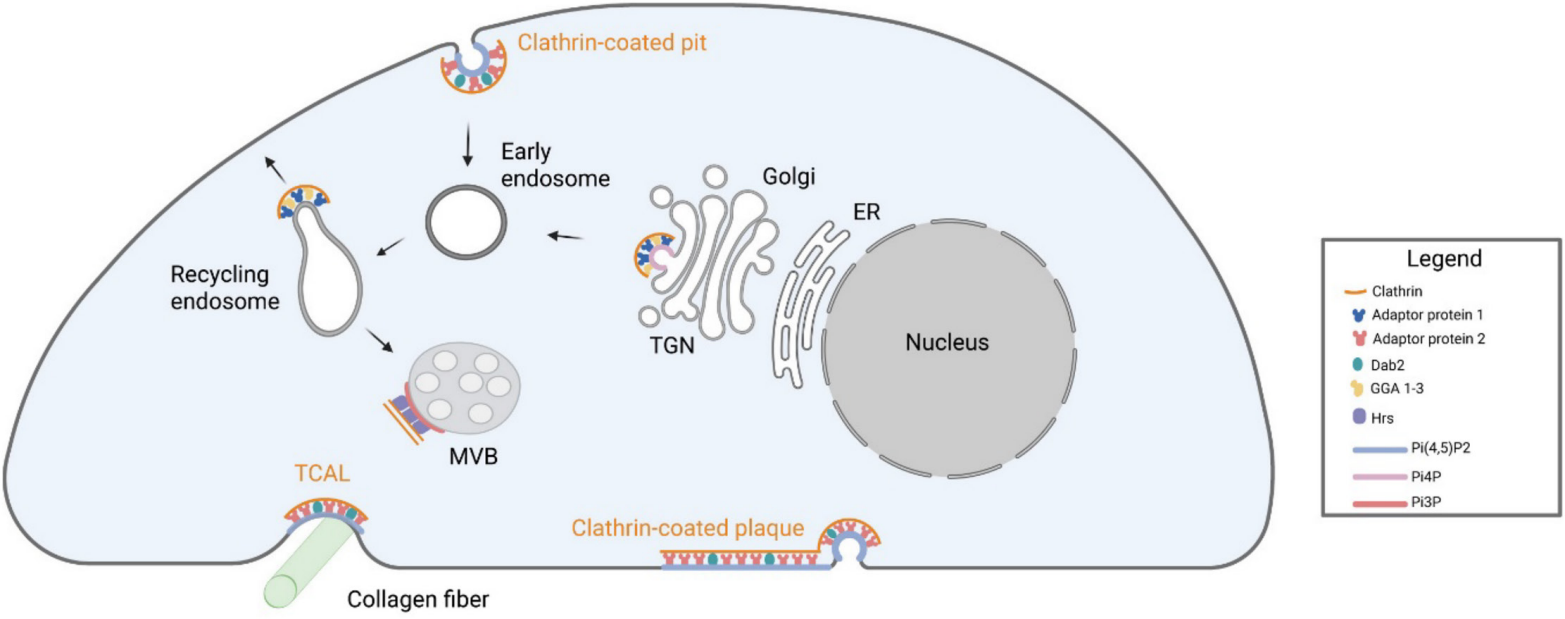
Structural diversity of clathrin assemblies across the endocytic and secretory pathways. Clathrin assembles into a wide range of membrane‐associated structures that support cargo sorting, membrane trafficking, adhesion, and extracellular matrix remodeling. At the plasma membrane, clathrin forms canonical clathrin‐coated pits (CCPs) through interactions with AP2 and Dab2 adaptors and PI(4,5)P_2_‐enriched domains, driving receptor‐mediated endocytosis. Larger and more stable clathrin‐coated plaques also form at the cell surface, where they contribute to adhesion and mechanotransduction. Specialized flat clathrin lattices known as tubular clathrin/AP2 lattices (TCALs) associate with collagen fibres and mediate cell attachment to extracellular matrix tracks. Within the endocytic pathway, clathrin localizes to recycling endosomes and multivesicular bodies (MVBs), where distinct adaptor and phosphoinositide compositions regulate cargo sorting and membrane remodeling. At the trans‐Golgi network (TGN), clathrin is recruited by AP1 and GGA adaptors on PI4P‐rich membranes to generate transport carriers destined for endosomal compartments.

The studies discussed here reveal that clathrin assemblies adopt diverse architectures adapted to the physical and functional constraints of specific membrane domains. Beyond canonical coated vesicles, clathrin can form stable planar lattices, tubular assemblies, and highly dynamic sorting platforms specialized for adhesion, mechanotransduction, and compartmental organization (summarized schematically in Figure 5).

A particularly instructive comparison emerges between clathrin assemblies in neurons and in skeletal muscle. In both systems, clathrin forms long‐lived, non‐canonical structures that are poorly productive for endocytosis yet essential for maintaining membrane organization under extreme physiological constraints. At the axon initial segment, clathrin assembles into stable, spatially confined curved pits that are positioned between cytoskeletal clearings and can persist for extended periods before undergoing stimulus‐dependent internalization. In skeletal muscle, clathrin plaques form extensive flat lattices that anchor adhesion complexes and reinforce the sarcolemma against contractile stress. Despite these clear differences in geometry, both systems illustrate how clathrin assemblies can be stabilized by their local cytoskeletal environment and adapted to the specific structural and mechanical constraints of highly specialized membrane domains.

Thus, clathrin's capacity to form stable scaffolds is not a specialized adaptation restricted to a single tissue, but a conserved solution that can be independently deployed in polarized or mechanically stressed domains.

From a pathological perspective, this expanded view of clathrin function has important implications. In neurons, disruption of axon initial segment organization is associated with defects in excitability and polarity. Given the emerging role of clathrin‐mediated endocytosis in maintaining membrane compartmentalization at the AIS, perturbations of AIS‐associated clathrin assemblies may contribute to these phenotypes, although direct evidence remains limited. Moreover, clathrin‐mediated endocytosis is essential for synaptic vesicle recycling and sustained neurotransmission [112]. A growing body of evidence implicates defects in endocytic trafficking in the pathogenesis of neurodegenerative disorders, including Alzheimer's disease through alterations in PICALM‐ and Amphiphysin 2/Bin1‐dependent pathways and Parkinson's disease through mutations affecting endocytic regulators such as DNAJC6, SYNJ1, and LRRK2 [127, 128]. In skeletal muscle, mutations in dynamin 2 (DNM2) cause the autosomal dominant form of centronuclear myopathy (CNM). Similarly, aberrant alternative splicing of *CLTC* and *BIN1* drives the pathophysiology of myotonic dystrophy type 1 (DM1). Together, these conditions underscore the critical role of clathrin‐associated trafficking pathways in neuromuscular disease. Compromised clathrin plaque formation or stability could weaken membrane‐cytoskeleton coupling, predisposing fibres to membrane damage and degeneration [13, 93, 94]. Recognizing these scaffold‐like and mechanoadaptive functions of clathrin will therefore be important for linking molecular defects to tissue‐specific pathology.

Looking forward, several challenges and opportunities emerge. A key priority will be to determine how cells regulate transitions between different clathrin architectures and whether such transitions occur dynamically in response to changes in mechanical load, signaling state, or developmental stage. Another open question concerns how distinct clathrin assemblies compete for or coordinate shared pools of clathrin and adaptors. Addressing these issues will require integrative approaches that combine quantitative imaging, ultrastructural analysis, and mechanical perturbation across multiple model systems.

Clathrin should be viewed not merely as a vesicle coat but as a structural scaffold capable of supporting trafficking, sorting, adhesion, and membrane stabilization. By viewing clathrin structural plasticity as a central organizing principle, it becomes possible to reconcile a wide range of observations across cell types and compartments within a coherent conceptual model. This perspective not only enriches our understanding of membrane trafficking but also positions clathrin as a key integrator of cellular organization.

## Funding

This work was supported by Agence Nationale de la Recherche (ANR‐21‐CE13‐0018 and ANR‐21‐CE11‐0009), Institut de Myologie, and Institut National de la Santé et de la Recherche Médicale.

## Conflicts of Interest

The authors declare no conflicts of interest.

## Data Availability

The data that support the findings of this study are available on request from the corresponding author. The data are not publicly available due to privacy or ethical restrictions.
